# Lightweight Statistical and Texture Feature Approach for Breast Thermogram Analysis †

**DOI:** 10.3390/jimaging11100358

**Published:** 2025-10-13

**Authors:** Ana P. Romero-Carmona, Jose J. Rangel-Magdaleno, Francisco J. Renero-Carrillo, Juan M. Ramirez-Cortes, Hayde Peregrina-Barreto

**Affiliations:** Instituto Nacional de Astrofísica, Óptica y Electrónica (INAOE) Tonantzintla, Puebla 72840, Mexico; ana.rcp31@gmail.com (A.P.R.-C.); jrangel@inaoe.mx (J.J.R.-M.); paco@inaoep.mx (F.J.R.-C.); jmram@inaoep.mx (J.M.R.-C.)

**Keywords:** thermography, breast cancer, image classification, machine learning

## Abstract

Breast cancer is the most commonly diagnosed cancer in women globally and represents the leading cause of mortality related to malignant tumors. Currently, healthcare professionals are focused on developing and implementing innovative techniques to improve the early detection of this disease. Thermography, studied as a complementary method to traditional approaches, captures infrared radiation emitted by tissues and converts it into data about skin surface temperature. During tumor development, angiogenesis occurs, increasing blood flow to support tumor growth, which raises the surface temperature in the affected area. Automatic classification techniques have been explored to analyze thermographic images and develop an optimal classification tool to identify thermal anomalies. This study aims to design a concise description using statistical and texture features to accurately classify thermograms as control or highly probable to be cancer (with thermal anomalies). The importance of employing a short description lies in facilitating interpretation by medical professionals. In contrast, a characterization based on a large number of variables could make it more challenging to identify which values differentiate the thermograms between groups, thereby complicating the explanation of results to patients. A maximum accuracy of 91.97% was achieved by applying only seven features and using a Coarse Decision Tree (DT) classifier and robust Machine Learning (ML) model, which demonstrated competitive performance compared with previously reported studies.

## 1. Introduction

Breast cancer is a malignant disease characterized by the uncontrolled growth of cancerous cells in breast tissue, leading to the formation of tumors. Over time, these tumors can invade surrounding tissues and spread to other parts of the body, a process known as metastasis [1]. Globally, breast cancer is the most commonly diagnosed cancer in women and represents the leading cause of death from malignant tumors. In 2020, approximately 685,000 deaths were attributed to this disease, making it the primary cause of cancer-related mortality among women [2,3]. Over the years, innovative techniques have been developed for the diagnosis and treatment of breast cancer, contributing to a reduction in the overall mortality rate. However, early detection remains one of the primary challenges in the healthcare field [4]. Early detection is crucial for the timely implementation of treatments and improving survival rates. Currently, the most widely used technologies for breast cancer detection include mammography, tomosynthesis, ultrasound, and magnetic resonance imaging [5]. However, the use of radiation and high costs are limitations that must be taken into consideration. These limitations have driven the development of new complementary tools to improve early breast cancer detection.

Over the past few decades, the use of infrared thermography in medical applications has been extensively studied. Thanks to advances in science and technology, the sensitivity of this technique has increased significantly, enabling it to detect subtle temperature variations [6]. The infrared radiation emitted by the body is captured through a thermal camera, allowing for the measurement of these thermal changes. Thus, thermography provides a fast, non-invasive, painless, and low-cost alternative [7,8,9]. Its use as a complementary diagnostic tool has been explored in various medical fields. In patients with rheumatic diseases, thermal changes were associated with areas of pain or swelling [10]. In the diagnosis and monitoring of peripheral vascular diseases that affect the peripheral circulatory system, it has been used to study varicose veins and diabetic foot [11]. In the context of respiratory diseases, thermography has proven helpful in identifying the intersegmental plane of the lungs, which facilitates pulmonary surgeries [12]. In oncology, it has been employed to detect thermal anomalies related to breast cancer, skin cancer, and metastasis of head and neck cancer, among others [13,14,15].

In 1956, Lawson identified a correlation between increased skin temperature and the presence of breast carcinoma, suggesting it as an indicator of anomalies associated with this disease. Cancerous cells require a greater supply of oxygen and nutrients to grow and propagate, which triggers a process called angiogenesis—the formation of new blood vessels around tumors. This increase in blood flow and metabolism within the tumor raises the temperature of the affected area compared with the surrounding tissues. As a result of these findings, the FDA approved thermography as a complementary tool for assessing the risk of breast tumors. According to the American College of Clinical Thermology, thermography has several advantages: The results are unaffected by breast tissue density, the presence of implants, or hormonal changes. Additionally, it is safe for continuous use, as it does not expose patients to radiation.

Nevertheless, interpreting thermographic images (thermograms) is challenging due to the wide variability in temperature distributions. To address this, computer vision and Machine Learning (ML) methods have been applied for anomaly detection, diagnosis, and surgical guidance [16]. In breast cancer research, ML, Deep Learning (DL), and Transfer Learning approaches have been used to classify thermograms as healthy or cancerous, improving diagnostic efficiency. These algorithms can support clinical decision-making and potentially enhance patient survival [17]. Recent studies have explored combining thermography with ML for breast cancer detection [18]. This approach can reduce human subjectivity by enabling automated interpretation of thermal images. However, many ML- or DL-based methods rely on a large number of features, which can limit interpretability for medical professionals. Thus, there is a need for approaches that use a smaller set of optimal features while maintaining classification accuracy.

This study proposes a descriptive framework to facilitate the interpretation of temperature information in breast thermograms. While elevated temperature and changes in its distribution are associated with abnormal behavior in the breast region, such variations do not consistently follow a spatial pattern, making their characterization challenging. To address this, statistical and textural features were computed, and the most informative ones were selected to provide a concise yet effective description of healthy and cancerous thermograms. Feature analysis was restricted to the breast region to minimize interference from surrounding anatomical areas, and each breast was evaluated individually, given the potential for intra-subject differences. The proposed description was evaluated using four classifiers, achieving an accuracy of 91.97%. Moreover, through the analysis of the 95% confidence intervals of the performance metrics using the bootstrap and McNemar tests, it was demonstrated that the proposed description remains consistent regardless of the classifier used. These results demonstrate that a lightweight feature set, combined with classical descriptors, enables the accurate classification of breast thermograms, offering a competitive alternative to current state-of-the-art methods.

## 2. Materials and Methods

### 2.1. Dataset

**Figure 1:**
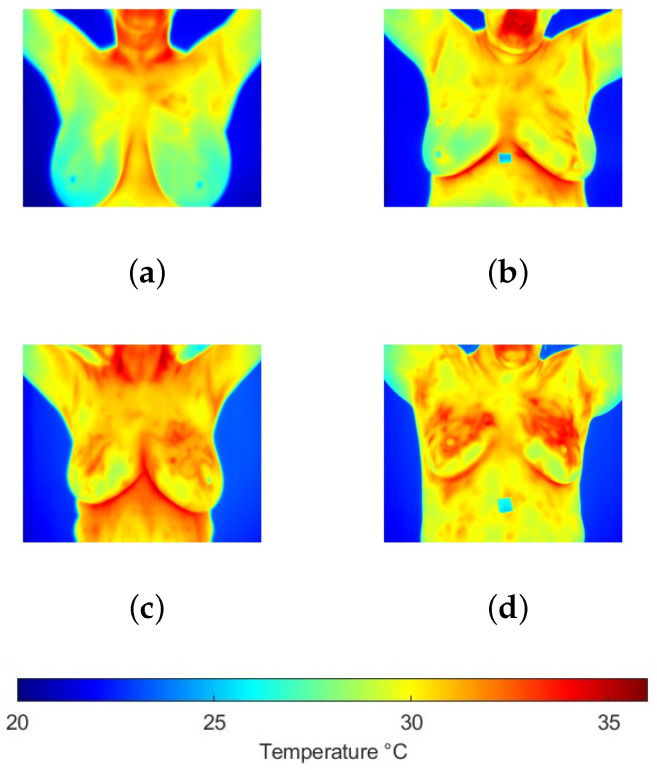
Breast thermograms samples from the (**a**,**b**) control and (**c**,**d**) cancer groups, where it is observed that increased temperatures are present in the second group.

To develop this work, a reference thermographic image database was used. The Dataset for Mastology Research (DMR) is a publicly available database developed by Federal Fluminense University in Brazil and has been widely referenced in the scientific literature. The DMR contains a set of thermographic images corresponding to patients classified as healthy or diseased. These images were captured using an FLIR SC-620 infrared camera, which offers a thermal sensitivity of 0.04 °C and a resolution of 640×480 pixels [19]. This database has been particularly used in research focused on the early detection of breast cancer, making it relevant for the present study. To mitigate redundancy and reduce the risk of overoptimistic bias in performance estimates, images with similar viewing angles and no significant differences were excluded from analysis. The evaluation was restricted to front-view images, which offer the most comprehensive visualization of the breast region. Furthermore, thermogram analysis was performed using temperature values extracted from radiometric files, as color palettes merely provide perceptual contrast without modifying the quantitative information. Taking these considerations into account, 141 thermograms were selected, of which 99 corresponded to the control group and 42 to the group of patients with cancer confirmed by biopsy. Figure 1 shows some samples of both groups. In order to maintain consistency in the representation of thermograms, the information is presented using the same temperature scale in a range of [20, 36] °C.

### 2.2. ROI Extraction

**Figure 2:**
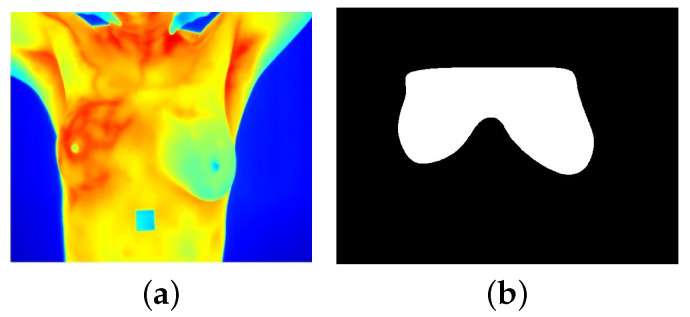
(**a**) Breast thermogram and (**b**) its corresponding binary mask.

In clinical thermograms, certain areas often exhibit elevated temperatures that do not necessarily indicate abnormality. For instance, the armpits and neck typically register higher temperatures compared with the breast region. However, in the context of breast temperatures, a similar pattern may indicate an abnormality. Therefore, it was decided to perform image segmentation to separate the regions of interest (ROIs) for analysis. During the segmentation process, a set of binary masks was manually created to separate the breast region from the background, allowing the application of specific local operations on the images. For each thermogram, a binary mask covering the area of both breasts was generated (Figure 2). Subsequently, each mask was divided into two by making a vertical division in the middle of both breasts to obtain two new masks corresponding to each breast individually. This decision was made after a series of tests revealed that analyzing the breasts individually provided a more precise approach and more robust results. This is because the thermal conditions of each breast can vary significantly; for example, some patients with temperature anomalies may present such behavior in only one breast.

### 2.3. Feature Extraction

One of the key processes for classification is image analysis, where it is essential to identify and quantify specific features that are relevant for accurate classification. By focusing solely on the data that most precisely describes the content of the image, the complexity of the process is reduced, allowing the analysis to focus on critical aspects such as edges, textures, shapes, and patterns, among others. In general, two main types of features can be extracted: statistical features and texture features. Statistical features describe the distribution of pixel values in the image, while texture features capture the variability and spatial patterns present in the image.

In this study, both types of features were employed to improve classification results by leveraging the relevant aspects that thermograms can contribute to this task. For feature selection, a thorough review of the existing literature related to the classification of thermographic images was conducted. The goal was to rigorously identify those features that could optimize the efficiency of the classification methods used. Based on the literature review, the following features were selected [20,21,22,23,24]:

(a) Statistical features

- Mean (μ): The average intensity value of the pixels that make up an image, indicating the mean color or brightness value in the image, where T(x,y) represents the temperature at position (x,y) in an image of size M×N.
  (1) $$\mu =\frac{1}{MN}\sum_{x=0}^{M-1}\sum_{y=0}^{N-1}T\left(x,y\right)$$
- Standard deviation (σ) and variance ($\sigma^{2}$): Indicate the dispersion of values in an image. Variance measures how much the pixel values deviate from the overall mean of these values in the image. The standard deviation, which is the square root of the variance, provides a more direct measure of dispersion. Both metrics offer insights into the variability of texture and contrast in the image.
  (2) $$\sigma =\sqrt{\frac{1}{MN}\sum_{x=0}^{M-1}\sum_{y=0}^{N-1}{\left(T\left(x,y\right)-\mu \right)}^{2}}$$
- Skewness (γ): A measure that indicates the degree of symmetry in the pixel distribution, with a particular emphasis on asymmetry. If the intensity values in an image are concentrated at one end of the spectrum, it suggests asymmetry, which may reveal specific patterns or features in the image.
  (3) $$\gamma =\frac{\mu -median}{\sigma }$$
- Kurtosis (*k*): A quantitative metric that characterizes the shape of the pixel distribution. It is utilized in image analysis to detect underlying patterns that may not be discernible through visual inspection.
  (4) $$k=\frac{E{\left(x-\mu \right)}^{4}}{\sigma^{4}}$$
- Entropy (*H*): A measure of randomness in the distribution of pixel values in an image. It is a tool that provides a representative value of the complexity in a specific section of the image, where *G* is the number of gray levels.
  (5) $$H=-\sum_{i=1}^{G}p_{i}\log_{2}\left(p_{i}\right)$$
- Maximum and minimum values: Values that represent the extremes of the range of pixel intensities in an image. In the case of thermographic images, the minimum value indicates the lowest intensity (temperature) detected, while the maximum value indicates the highest intensity.
- Coefficient of variation (CV): A measure that evaluates the deviations of pixels from their mean and the dispersion among them. In a thermographic image, the coefficient of variation indicates the variability of temperature in the image.
  (6) $$CV=\frac{\sigma }{\mu }\times 100$$

(b) Texture features

Features were obtained using the Gray-Level Co-occurrence Matrix (GLCM), which describes the frequency with which one gray level appears near another gray level within a small region of the image. In other words, the GLCM provides information about the distribution of gray levels relative to each other in a specific area of the image, thus allowing the observation of pixel clustering patterns [23,25].

For the construction of the co-occurrence matrices, gray levels were considered in the range of [0,255]. The co-occurrences between the central pixel and those at a distance of one were analyzed, considering four directions $\left\{0^{\circ },45^{\circ },90^{\circ },135^{\circ }\right\}$. The matrices obtained in each direction were integrated for an integral analysis of changes in all directions. From the resulting matrix, the properties of homogeneity, contrast, correlation, and energy were extracted as described below.

- Homogeneity: A measure that indicates how uniform the distribution of pixel values is within a region of an image. High homogeneity suggests that the gray levels in the image are very similar to each other.
  (7) $$\mathrm{Homogeneity}=\sum_{x,y}\frac{1}{1+{\left(x-y\right)}^{2}}T\left(x,y\right)$$
- Contrast: It measures the difference in intensity between the pixels of an image. A higher contrast indicates that there are significant differences in pixel intensities.
  (8) $$\mathrm{Contrast}=\sum_{x,y}{\left(x-y\right)}^{2}T\left(x,y\right)$$
- Correlation: A measure that describes the relationship between pixel values, helping to identify patterns or trends in their arrangement. High contrast indicates that the pixel values are uniformly distributed in the GLCM.
  (9) $$\mathrm{Correlation}=\frac{\sum_{x,y}\left(x-\mu_{x}\right)\left(y-\mu_{y}\right)T\left(x,y\right)}{\sigma_{x}\sigma_{y}}$$
- Energy (*E*): A measure that describes the variation in intensity between pixels, taking into account how factors such as color, brightness, magnitude, shadows, or textures change as one moves from one pixel to another. In an image, areas with edges or textured patterns contain higher energy, indicating greater visual complexity. In thermographic images, energy is calculated by summing the intensities (temperatures) of each pixel in the image, as shown in Equation (10).
  (10) $$E=\sum_{x=0}^{M-1}\sum_{y=0}^{N-1}T\left(x,y\right)$$

### 2.4. Feature Selection

To achieve a more efficient and effective classification process, feature reduction can be a valuable tool, as it helps confirm the relevance of each feature used. A statistical test was conducted to verify that the selected features provided meaningful information for image classification. The Student’s *t*-test was chosen, which is used to determine if there is a significant difference between the means of two groups [26]. In this context, it will be applied to identify features that genuinely help differentiate between images with a higher probability of cancer and those with more similarity to the control.

The first step in this process is to establish the null and alternative hypotheses. The null hypothesis states that there is no significant difference between the means of the two classes, while the alternative hypothesis suggests that there is a significant difference. Next, the data should be divided into two subsets corresponding to each of the two existing classes, labeled as $C_{1}$ and $C_{2}$, with samples being represented by $X_{1}$ and $X_{2}$, respectively. Finally, for each feature *i*, the mean in both classes is calculated using Equation (11).

(11) $$\begin{array}{c} {\bar{X}}_{i,C_{1}}=\frac{1}{n_{1}}\sum_{j=1}^{n_{1}}X_{i,j,C_{i}}\\ {\bar{X}}_{i,C_{2}}=\frac{1}{n_{2}}\sum_{j=1}^{n_{2}}X_{i,j,C_{2}} \end{array}$$

where ${\bar{x}}_{i,C_{1}}$ and ${\bar{x}}_{i,C_{2}}$ represent the mean of feature *i* in classes $C_{1}$ and $C_{2}$, respectively; $n_{1}$ and $n_{2}$ represent the number of samples in classes $C_{1}$ and $C_{2}$, respectively. $x_{i,j,C_{1}}$ and $x_{i,j,C_{2}}$ represent the value of feature *i* in sample *j* for classes $C_{1}$ and $C_{2}$, respectively.

To conduct this test, it is essential to calculate the mean (μ) and variance ($\sigma^{2}$) of each feature *i* in both classes. The t-statistic is determined for each feature i using Equation (12). Subsequently, using t-distribution tables, which provide critical values for the t-distribution at various significance levels and degrees of freedom, the corresponding *p*-value for each feature is determined. This *p*-value indicates whether the null hypothesis is accepted, meaning that there is no significant difference between the means of the two classes for that particular feature. In this study, a significance level of 0.05 was used, so features with a *p*-value lower than the established significance level are considered relevant and useful for classification [27].

(12) $$t_{i}=\frac{{\bar{X}}_{i,C_{1}}-{\bar{X}}_{i,C_{2}}}{\sqrt{\frac{\sigma_{1i}^{2}}{n_{1}}+\frac{\sigma_{2i}^{2}}{n_{2}}}}$$

### 2.5. Model Validation

To evaluate the performance of classifiers, a stratified cross-validation was employed to assess the replicability of the results, as well as the generalization and stability of the findings. This approach has proven helpful for imbalanced datasets since a purely random partition could lead to folds with insufficient representation of the minority class [28]. In stratified cross-validation, the dataset is divided into k folds in a way that preserves the overall class distribution of the complete dataset within each fold. For example, if the dataset contains 80% of samples from class 0 and 20% from class 1, each fold will approximately maintain this same proportion.

**Figure 3:**
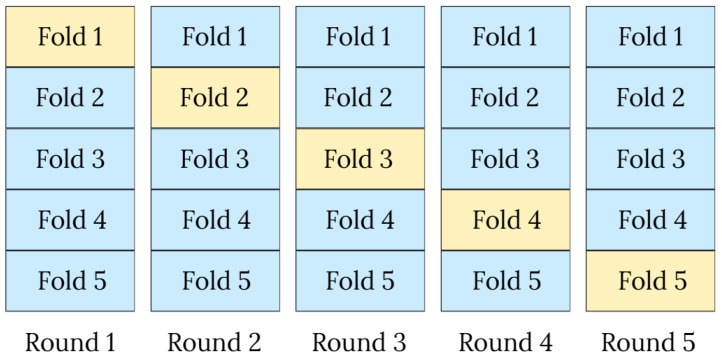
Illustration of the stratified 5-fold cross-validation procedure, where each fold is used once as the test set (yellow) while the remaining folds (blue) are used for training.

At each iteration, k − 1 folds are selected for training, and the remaining fold is used for testing. Thus, in each round, a model is trained and fitted so that the training and test folds differ each time, as illustrated in Figure 3. This strategy enables the representation of both majority and minority classes in each fold, thereby reducing variance and bias in performance metrics.

### 2.6. Classification Methods

Although CNNs or DL models have also been employed in this context, they require extensive samples to achieve robust learning and avoid overfitting [29]. Particularly, in medical datasets, the scarcity of labeled data along with the importance of clinical variability poses an important consideration for applying these models. On the other hand, traditional ML is an appropriate approach when there is a limited set of data available [30,31,32]. To accomplish this classification task, four ML models were employed: SVM (quadratic and linear), LR, and DT.

The Support Vector Machine (SVM) is a supervised learning algorithm that operates by mapping data to a high-dimensional feature space using kernel functions, enabling the classification of data into distinct categories. SVM aims to identify a hyperplane that separates the data of different classes within the feature space, choosing the hyperplane that maximizes the minimum distance between the hyperplane itself and the closest data points from each class. In this study, two variants of the SVM algorithm were utilized: linear SVM and quadratic SVM. The primary distinction between them lies in the shape of the hyperplane used to separate the classes, which could be a straight line or a quadratic curve [33]. The Logistic Regression (LR) algorithm performs binary classification tasks by predicting the probability of an outcome or observation expressed in a binary value. It is widely used in predictive models, where it estimates the likelihood that an instance belongs to a specific category or not. For the purposes of this study, the categories will be associated with thermograms that have or do not have [34,35] them. The Coarse Tree (CT) method is a supervised learning approach based on DT that employs iterative segmentation of the feature space to create simpler regions. Additionally, it tends to generalize more, which reduces the likelihood of overfitting the training data [36].

## 3. Experimental Results

**Figure 4:**
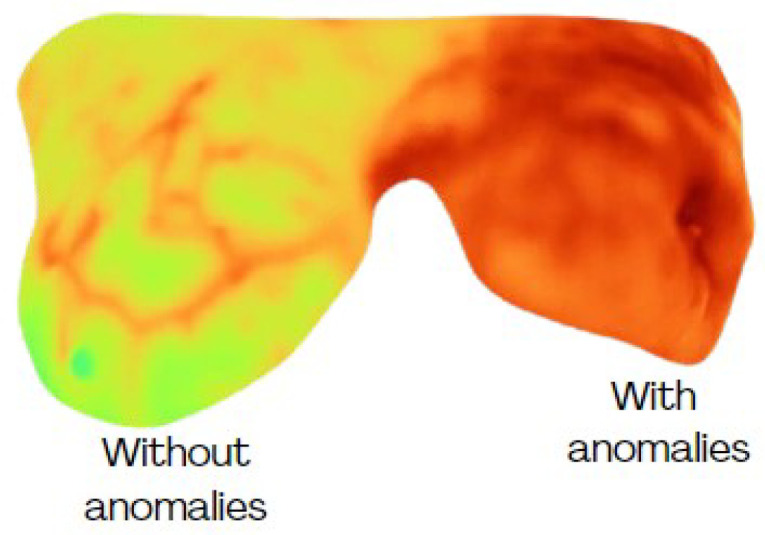
Different conditions of temperature among breasts from the same subject.

Thermograms were analyzed by focusing on the breast region only, i.e., extracting the area indicated by the binary mask (Figure 2). Nevertheless, a high degree of variability can be present among the breasts of the same subject (Figure 4), which can affect their evaluation. Therefore, this study considers each breast as an individual case. Some individual samples were removed when they presented low resolution (high blurring) or a breast amputation had occurred. A total of 265 individual thermographic samples were generated, comprising 71 samples from the cancer group and 194 from the control group. For model validation, a stratified 5-fold cross-validation was applied to test each classifier.

For feature selection, the Student’s *t*-test was applied to the 14 initially selected features, considering as optimal those with a *p*-value < 0.05. According to the *p*-value texture features of homogeneity, contrast, and correlation and four statistical features of entropy, min, and max values, were selected from Table 1. These seven features were used as input variables in the previously mentioned classifiers.

To assess the usefulness of the selected features and the performance of the classifiers that use them to classify thermograms, the evaluation metrics of precision, accuracy, recall, and F1-score (Ecs. 13–16) were used, where True Positives (TP), True Negatives (TN), False Positives (FP), and False Negatives (FN) are utilized.

(13) $$Accuracy=\frac{TP+TN}{TN+FP+FN+TP}$$

(14) $$Precision=\frac{TP}{FP+TP}$$

(15) $$Recall=\frac{TP}{FN+TP}$$

(16) $$F1\text{-}\mathrm{score}=\frac{TP}{TP+\frac{1}{2}\left(FP+FN\right)}$$

The classification results for each model used are presented in Table 2. The comparative evaluation shows that the CT achieved the best overall performance, attaining the highest accuracy (91.97%± 0.015), precision (90.79%± 0.025), and F1-score (92.09%± 0.014). The Q-SVM delivered the best recall (94.37%± 0.042), with competitive accuracy (90.99%± 0.019) and F1-score (91.256%± 0.020). LR obtained a precision of 85.04%± 0.019 but a lower recall (84.79%± 0.034), resulting in an accuracy of 84.93%± 0.020 and an F1-score of 84.89%± 0.022. The L-SVM yielded the lowest metrics overall (accuracy 84.93%± 0.023, precision 83.43%± 0.029, recall 87.32%± 0.042, and F1-score 85.27%± 0.024). Overall, CT provided the most balanced performance, while Q-SVM excelled in sensitivity, consistent with its ability to model non-linear decision boundaries via the quadratic kernel. Importantly, CT’s top accuracy/F1 with tight confidence bounds (±0.015/±0.014), together with its interpretability and low computational cost, indicates a robust and practical model for deployment in breast thermogram classification.

To perform a robust evaluation of classifier performances, confidence intervals (CIs) were calculated using bootstrap and McNemar analysis, which are particularly suited for assessing small datasets. Bootstrapping allows for the estimation of the variability of a metric without relying on parametric assumptions. By repeatedly sampling with replacement from the observed data, it constructs an empirical distribution of the statistic of interest and derives confidence intervals directly from it [37]. McNemar CIs, on the other hand, assess whether differences in predictions between paired classifiers are statistically significant [38,39].

A bootstrap procedure was applied with B=2000 replications, a value commonly recommended in the literature to ensure stable estimation of confidence intervals [37,40]. The evaluation was conducted on the four classification models and their performance metrics. For each model–metric combination, the empirical distribution of the bootstrap replications was used to compute the mean and the 95% percentile-based CI. The results in Table 3 present the mean performance metrics with 95% bootstrap confidence intervals for the four classifiers. Overall, CT and Q-SVM consistently achieved higher accuracy, precision, recall, specificity, and F1-score compared with L-SVM and LR, for which CIs are slightly wider. The narrow CIs for CT and Q-SVM indicate a more stable and reliable performance across resampled datasets, which suggests that the proposed description performs most robustly with these classifiers, providing high sensitivity and precision.

The McNemar test was applied to compare classifier performance on the same dataset, assessing whether the number of discordant predictions differed significantly between paired classifiers. Table 4 reports the mean difference in discordant predictions, the minimum and maximum 95% CI bounds across folds, and the associated *p*-values. No statistically significant differences were observed among the four classifiers. Although some pairs showed moderate mean differences (e.g., L-SVM vs. LR: 0.60), all 95% CIs included 0 and all *p*-values exceeded 0.05, indicating statistically equivalent performance across folds. This result indicates that the proposed descriptor can maintain consistency regardless of the classifier used, which could be helpful for generalization to other datasets.

**Figure 5:**
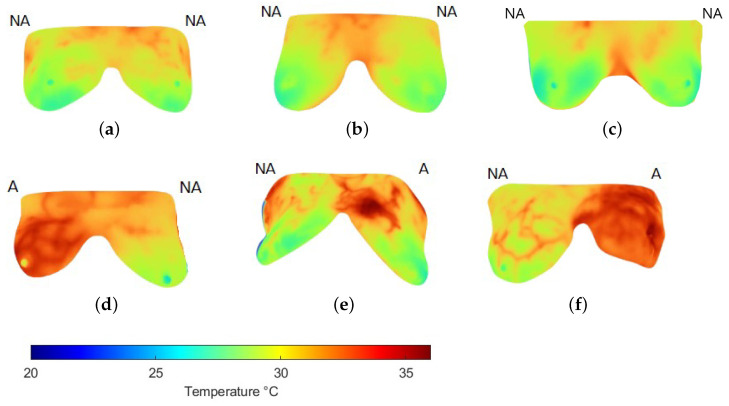
Examples of the breast region extraction in thermograms (**a**–**c**) of the control and (**d**–**f**) cancer groups. Labels above each breast indicate whether no anomalies (NA) or anomalies (A) were detected using the CT classifier, matching the expert’s label.

Figure 5 shows some samples of thermograms from the control group where the CT classifier, with the best performance according to the previous analysis, detected no anomalies (NAs) and where a high degree of symmetry in temperature distribution was observed. Although some subtle changes are perceived, they do not represent a significant difference. Nevertheless, in the samples identified with anomalies (As), the difference in thermal distribution is higher with increased temperatures in the breast with cancer confirmed by biopsy. Additionally, changes in vascularization have been identified in surrounding areas where the tumor develops as a result of the angiogenesis process (Figure 5d–f).

To complement these observations, Table 5 reports selected feature values for a control and a cancer case. Figure 5a illustrates a control example in which both breasts were labeled NA. Although their mean temperature values are similar, the right breast exhibits a broader, warmer region with slight vascularization, resulting in higher entropy. This localized change also produces noticeable differences in minimum and maximum values compared with the left breast, while correlation decreases. However, homogeneity and contrast remain close across both sides, as each breast exhibits relatively regular inner regions. These findings suggest that, even in NA cases from the same subject, the features can capture subtle variations not apparent to visual inspection.

In contrast, Figure 5d corresponds to a subject with cancer confirmed in the left breast, while the right breast was labeled as healthy. Here, mean, minimum, and maximum temperatures are all higher than in the control example. More evident vascularization is also observed in the right breast, reflected in reduced homogeneity. Because elevated temperatures extend across large areas in both breasts, correlation values remain high. Entropy and contrast also increase, with the latter showing greater sensitivity to thermal changes. Altogether, these characteristics enabled the CL classifier to discriminate between normal and abnormal patterns in thermal images effectively.

These labels were aligned with the annotations provided by the experts in the database, thereby validating the classification accuracy.

## 4. Discussion

Some works have addressed this task under diverse approaches. Sathish et al. [41] initially selected 144 spectral features for thermography classification, achieving an accuracy of 87% with the Bagged Trees (BTs) classifier and 5-fold cross-validation. Subsequently, feature reduction and selection techniques were applied, reducing the number of features to 33. This optimization improved the classification accuracy to 93% when using Ensemble Bagged Trees. Similarly, Lennox et al. [42] employed a dataset of 60 thermograms and 19 selected features, achieving a maximum accuracy of 90% with the Random Forest classifier. This study utilized a Leave-One-Out Cross-Validation scheme, highlighting its utility in evaluating model robustness and reliability. Kumar et al. [43] used a larger dataset of 200 thermograms and 20 selected features, achieving a maximum accuracy of 92.70% with the DT classifier. Other works have considered avoiding redundancy in exhaustive feature extraction. In [44], several feature selection strategies were applied, obtaining a reduced description down to six features and performing classification using an Artificial Neural Network (ANN). Additionally, this description was tested over thermograms from different groups considering ages between 20 and 80, obtaining accuracy ranging between 79.3 and 74.7%; the overall performance reached an accuracy of 88.57%. Recently, Mirasbekov et al. [45] analyzed a dataset of 364 thermograms (153 cancer, 211 healthy) using a CNN and Transfer Learning. The accuracy achieved was 90.93%.

The application of CNN and DL in thermography classification has been widely studied due to its potential advantages. However, its effectiveness depends on several critical factors. First, these models require large and diverse datasets to achieve robust training; otherwise, they are prone to overfitting and limited generalization [46]. This issue is particularly relevant in medical imaging, where data availability is often restricted. Indeed, some studies have reported that simpler ML models can outperform DL approaches in breast cancer detection, offering more reliable and practical results [47]. Consequently, the limitations of DL—especially its sensitivity to dataset size and variability—may hinder its performance in real-world applications or when new datasets are introduced. Feature selection significantly influences classification outcomes, and studies indicate that more cautious selection methods can yield better accuracy than exhaustive extraction techniques [48,49,50]. The CT classifier yielded the best results with the proposed description, reaching an accuracy of 91.97%.

Related works shown in Table 6 reveal that the number of features employed by researchers vary significantly. In the classification of breast thermographies, feature selection has been considered a relevant step to reduce computational costs and redundant features, helping to avoid overfitting and improve performance. In the present study, ML models were selected as a viable option due to the limitations associated with DL approaches, particularly the need for large datasets. This decision was validated by the results obtained, which demonstrated competitive performance compared with previous studies. A key advantage of this approach was the use of a reduced set of features, which not only optimized the model’s performance but also underscored the potential of ML techniques in achieving robust outcomes with limited data. By focusing on proper feature selection and modeling, this study advanced the field by illustrating that effective and reliable classification can be achieved without the extensive computational and data requirements of DL. This reinforces the critical importance of tailoring the model selection process to align with the specific characteristics and constraints of the problem being addressed and highlights the effectiveness of streamlined approaches in delivering reliable and efficient solutions in thermographic image classification.

The implementation of thermography has been extensively evaluated by various authors, who have obtained results supporting its potential as an effective complementary tool for detecting anomalies associated with breast cancer. This technique stands out for its ability to provide relevant information not observable through conventional methods. The results obtained in this work have proved that it is possible to achieve high-performance results with a lightweight description. This description was able to distinguish among the samples that presented significant thermal differences, which is consistent with the expert classification of healthy/cancer. Moreover, the applied tests provide a more reliable and rigorous framework for validating results, ensuring that the reported performance accurately reflects the true model behavior rather than random variation. The obtained CIs showed that the proposed description remains highly stable despite the used classifier.

Despite these promising results, some limitations remain. The results were obtained on a dataset of limited size. A larger and different dataset is required for external validation and generalization. Also, the analysis is limited to a binary identification of healthy and cancer samples. Not only a larger dataset but also a more precise labeling that includes intermediate classes or a possible association with the standard BI-RADS grades is required. Therefore, a continuous improvement in the research of thermal images for detecting risk cancer is necessary.

## 5. Conclusions

Through studies analyzing the functionality and utility of clinical thermography, it has been demonstrated that this is a valuable complementary tool for evaluating anomalies related to breast cancer, as skin temperature has been established as a sign of disease. Furthermore, these thermal changes are often imperceptible to the naked eye. However, some of these thermal changes may be so subtle that they are not visible to the naked eye, which underscores the need for automated models capable of precise analysis and accurate classification of thermographic features.

In this study, ML models were employed, demonstrating competitive accuracy in the individual analysis of each breast. This was achieved through feature selection and testing several classifiers, a critical aspect consistent with previous reports, as proper feature selection leads to better classification results. The results showed that an accuracy of 91.97% can be achieved, outperforming previous studies and competing with sophisticated current approaches, while avoiding exhaustive feature extraction. Moreover, a brief description enables the correlation of relevant features with clinical observations, thereby increasing the interpretability of the description. The proposed approach not only aligned with expert classifications but also proved to be stable across classifiers, as confirmed through statistical testing and the analysis of confidence intervals. This study demonstrated that selected ML models can accurately classify breast thermograms, offering a short description that can be used to complement standard diagnostic methods.

As future work, this hypothesis will be addressed along with the exploration of hybrid models to improve results. Moreover, expanding the current database, automating the masks generation, and incorporating new features from other domains is a worthwhile area for future investigation.

## Figures and Tables

**Table 1:** Student’s *t*-test *p*-value of the analyzed features.

Feature	*p*-Value
Homogeneity	0.0295
Contrast	0.0000
Correlation	0.0009
*E*	0.1408
μ	0.0000
σ	0.4762
$\sigma^{2}$	0.9625
γ	0.3051
*k*	0.3477
*H*	0.0018
Min	0.0018
Max	0.0000
CV	0.4189

**Table 2:** Classification results of breast thermograms considering a five-fold cross-validation.

Metrics	L-SVM	Q-SVM	LR	CT
Accuracy	84.93%± 0.023	90.99%±0.019	84.93%±0.020	91.97%±0.015
Precision	83.43%±0.029	88.41%±0.011	85.04%±0.019	90.79%±0.025
Recall	87.32%±0.042	94.37%±0.042	84.79%±0.034	93.52%±0.027
F1-score	85.27%±0.024	91.256%±0.020	84.89%±0.022	92.09%±0.014

**Table 3:** Mean performance metrics evaluation with their 95% bootstrap CIs for breast thermogram classification.

Metrics	L-SVM	Q-SVM	LR	CT
Accuracy	84.92 [82.25–87.46]	90.95 [88.87–93.10]	84.96 [82.39–87.61]	91.97 [90.00–93.94]
Precision	83.28 [80.15–86.38]	88.41 [85.53–91.20]	85.10 [82.07–88.12]	90.74 [88.14–93.34]
Recall	87.31 [83.94–90.70]	94.36 [91.83–96.62]	84.85 [81.13–88.45]	93.50 [90.70–95.77]
Specificity	82.60 [78.59–86.48]	87.62 [84.23–90.99]	85.12 [81.41–88.73]	90.43 [87.04–93.52]
F1-score	85.28 [82.58–87.74]	91.30 [89.18–93.19]	84.91 [82.16–87.61]	92.08 [90.16–93.98]

**Table 4:** Pairwise comparison of classifier performance using McNemar’s test.

Pair	Difference	95% CI	*p*-Value
L-SVM vs. Q-SVM	−0.10	[−0.72,0.57]	1.00
L-SVM vs. LR	0.60	[−0.69,0.99]	1.00
L-SVM vs. CT	0.07	[−0.62,0.67]	1.00
Q-SVM vs. LR	0.33	[−0.42,0.92]	1.00
Q-SVM vs. CT	0.25	[−0.73,0.95]	1.00
LR vs. CT	−0.16	[−0.74,0.51]	1.00

**Table 5:** Feature description of thermograms in Figure 5.

Name	Label	Mean	Entropy	Min	Max	Homogeneity	Contrast	Correlation
Figure 5a-L	NA	27.5098	0.6969	23.46	31.86	0.6286	4.0095	0.9922
Figure 5a-R	NA	27.6746	0.7346	21.84	32.59	0.6433	3.9272	0.9912
Figure 5d-L	A	31.5911	0.7130	23.13	33.57	0.6639	4.4011	0.9896
Figure 5d-R	NA	29.1874	0.7506	22.75	33.63	0.7048	3.4418	0.9950

**Table 6:** Summary of studies on the use of thermography and the evaluation of AI techniques for the early detection of breast cancer.

Work	Classification Model	Samples	Features	Balanced Classes	Accuracy
Sathish et al. [41] (2018)	BT Ensemble BT	-	33	-	87.00% 71.00%
Lennox et al. [42] (2021)	Random Forest ANN SVM RBF	60	19	Yes	90.00% 88.33% 88.33%
Kumar et al. [43] (2022)	DT KNN LDA	200	20	Yes	92.70% 91.00% 84.80%
Gupta et al. [44] (2023)	ANN	278	6	-	88.57%
Mirasbekov et al. [45] (2024)	CNN Transfer Learning	364	-	Yes	90.93%
Proposed	CT	141	7	Yes	91.97%

## Data Availability

The dataset analyzed in this study belongs to the DMR—Database For Mastology Research at https://visual.ic.uff.br/dmi/(accessed on 8 October 2025), which was previously reported in [19].
